# Dispelling the Myth: Training in Education or Neuroscience Decreases but Does Not Eliminate Beliefs in Neuromyths

**DOI:** 10.3389/fpsyg.2017.01314

**Published:** 2017-08-10

**Authors:** Kelly Macdonald, Laura Germine, Alida Anderson, Joanna Christodoulou, Lauren M. McGrath

**Affiliations:** ^1^Department of Psychology, University of Houston Houston, TX, United States; ^2^Department of Psychiatry, McLean Hospital, Harvard Medical School Belmont, MA, United States; ^3^School of Education, American University Washington, DC, United States; ^4^Communication Sciences and Disorders, MGH Institute of Health Professions Charlestown, MA, United States; ^5^Department of Psychology, University of Denver Denver, CO, United States

**Keywords:** neuromyths, educational neuroscience, learning styles, dyslexia

## Abstract

Neuromyths are misconceptions about brain research and its application to education and learning. Previous research has shown that these myths may be quite pervasive among educators, but less is known about how these rates compare to the general public or to individuals who have more exposure to neuroscience. This study is the first to use a large sample from the United States to compare the prevalence and predictors of neuromyths among educators, the general public, and individuals with high neuroscience exposure. Neuromyth survey responses and demographics were gathered via an online survey hosted at TestMyBrain.org. We compared performance among the three groups of interest: educators (*N* = 598), high neuroscience exposure (*N* = 234), and the general public (*N* = 3,045) and analyzed predictors of individual differences in neuromyths performance. In an exploratory factor analysis, we found that a core group of 7 “classic” neuromyths factored together (items related to learning styles, dyslexia, the Mozart effect, the impact of sugar on attention, right-brain/left-brain learners, and using 10% of the brain). The general public endorsed the greatest number of neuromyths (*M* = 68%), with significantly fewer endorsed by educators (*M* = 56%), and still fewer endorsed by the high neuroscience exposure group (*M* = 46%). The two most commonly endorsed neuromyths across all groups were related to learning styles and dyslexia. More accurate performance on neuromyths was predicted by age (being younger), education (having a graduate degree), exposure to neuroscience courses, and exposure to peer-reviewed science. These findings suggest that training in education and neuroscience can help reduce but does not eliminate belief in neuromyths. We discuss the possible underlying roots of the most prevalent neuromyths and implications for classroom practice. These empirical results can be useful for developing comprehensive training modules for educators that target general misconceptions about the brain and learning.

## Introduction

Educational neuroscience (also known as mind, brain, education science) is an emerging field that draws attention to the potential practical implications of neuroscience research for educational contexts. This new field represents the intersection of education with neuroscience and the cognitive and developmental sciences, among other fields, in order to develop evidence-based recommendations for teaching and learning (Fischer et al., 2010). This emerging field has garnered growing interest (i.e., Gabrieli, 2009; Carew and Magsamen, 2010; Sigman et al., 2014), but it is also widely recognized that attempts to create cross-disciplinary links between education and neuroscience may create opportunities for misunderstanding and miscommunication (Bruer, 1997; Goswami, 2006; Bowers, 2016). In the field of educational neuroscience, some of the most pervasive and persistent misunderstandings about the function of the brain and its role in learning are termed “neuromyths” (OECD, 2002).

The Brain and Learning project of the UK's Organization of Economic Co-operation and Development (OECD) drew attention to the issue of neuromyths in 2002, defining a neuromyth as “a misconception generated by a misunderstanding, a misreading, or a misquoting of facts scientifically established (by brain research) to make a case for the use of brain research in education or other contexts” (OECD, 2002). Neuromyths often originate from overgeneralizations of empirical research. For example, the neuromyth that people are either “left-brained” or “right-brained” partly stems from findings in the neuropsychological and neuroimaging literatures demonstrating the lateralization of some cognitive skills (i.e., language). The fact that some neuromyths are vaguely based on empirical findings that have been misunderstood or over-exaggerated can make some neuromyths difficult to dispel.

There are several factors that contribute to the emergence and proliferation of neuromyths, most notably: (1) differences in the training background and professional vocabulary of education and neuroscience (Howard-Jones, 2014), (2) different levels of inquiry spanning basic science questions about individual neurons to evaluation of large-scale educational policies (Goswami, 2006), (3) inaccessibility of empirical research behind paywalls which fosters increased reliance on media reports rather than the original research (Ansari and Coch, 2006), (4) the lack of professionals and professional organizations trained to bridge the disciplinary gap between education and neuroscience (Ansari and Coch, 2006; Goswami, 2006), and (5) the appeal of explanations that are seemingly based on neuroscientific evidence, regardless of its legitimacy (McCabe and Castel, 2008; Weisberg et al., 2008).

Many leaders in the field have pointed out the potential benefits of bidirectional collaborations between education and neuroscience (Ansari and Coch, 2006; Goswami, 2006; Howard-Jones, 2014), but genuine progress will require a shared foundation of basic knowledge across both fields. One first step in this pursuit should be dispelling common neuromyths. Toward this end, we launched the current study to identify and quantify neuromyths that persist in educational circles and to test whether these myths are specific to educators or whether they persist in the general public and in those with high exposure to neuroscience as well. The goal of this study was to provide empirical guidance for teacher preparation and professional development programs.

The existence of neuromyths has been widely acknowledged in both the popular press (i.e., Busch, 2016; Weale, 2017) and in the educational neuroscience field (Ansari and Coch, 2006; Goswami, 2006, 2008; Geake, 2008; Pasquinelli, 2012), which has prompted research efforts to quantify teachers' beliefs in neuromyths across countries and cultures. Most of these studies suggest that the prevalence of neuromyths among educators may be quite high (Dekker et al., 2012; Simmonds, 2014). For example, Dekker et al. (2012) administered a neuromyths survey to primary and secondary school teachers throughout regions of the UK and the Netherlands and found that, on average, teachers believed about half of the myths (Dekker et al., 2012). Persistent neuromyths included the idea that students learn best when they are taught in their preferred learning style (i.e., VAK: visual, auditory, or kinesthetic; Coffield et al., 2004; Pashler et al., 2008), the idea that students should be classified as either “right-brained” or “left-brained,” and the idea that motor coordination exercises can help to integrate right and left hemisphere function. Surprisingly, they found that educators with more general knowledge about the brain were also more likely to believe neuromyths (Dekker et al., 2012). This finding that more general brain knowledge was related to an increased belief in neuromyths is surprising. One potential explanation is that teachers who are interested in learning about the brain may be exposed to more misinformation and/or may misunderstand the content such that they end up with more false beliefs. However, it is equally possible that teachers who believe neuromyths may seek out more information about the brain.

To date, the largest study of educators (*N* = 1,200) was conducted in the UK by the Wellcome Trust using an online survey of neuromyths (Simmonds, 2014). Consistent with the results of Dekker et al. (2012), Simmonds et al. found that the learning styles neuromyth was the most pervasive with 76% of educators indicating that they currently use this approach in their practice. The next most frequently endorsed neuromyth was left/right brain learning with 18% of educators reporting that they are currently using this idea in their practice (Simmonds, 2014). These results clearly indicate that neuromyths continue to persist among educators and are being used in current practice.

Additional studies utilizing similar surveys of neuromyths have been conducted with samples of teachers in Greece (Deligiannidi and Howard-Jones, 2015), Turkey (Karakus et al., 2015), China (Pei et al., 2015), and Latin America (Gleichgerrcht et al., 2015). Similar patterns of neuromyth endorsement have emerged across this body of literature. Two of the most pervasive myths across countries have been related to learning styles and left-brain/right-brain learning.

The global proliferation of neuromyths among educators is concerning as many of the neuromyths are directly related to student learning and development, and misconceptions among educators could be deleterious for student outcomes. For example, if an educator believes the myth that dyslexia is caused by letter reversals, students who have dyslexia but do not demonstrate letter reversals might not be identified or provided appropriate services. Another harmful consequence of neuromyths is that some “brain-based” commercialized education programs are based on these misconceptions and have limited empirical support. School districts that are unfamiliar with neuromyths may devote limited time and resources to such programs, which could have otherwise been used for empirically-validated interventions. Thus, it is important to obtain additional data about the prevalence and predictors of neuromyths in order to design effective approaches for dispelling these myths.

Available studies examining the prevalence of neuromyths provide information about neuromyth endorsement from a range of countries and cultures; however, no study to date has compared teachers' beliefs in neuromyths to a group of non-educators. Furthermore, to our knowledge, no study has systematically examined neuromyths in a sample from the United States. Given large variations in teacher preparation across countries, it is worthwhile to explore the prevalence of neuromyths in a US sample. For this reason, the current study recruited a US sample of educators and included a comparison group of individuals from the general public. We also included a second comparison group of individuals with high neuroscience exposure to further contextualize the results from the groups of educators and general public. Notably, most previous studies have used samples of educators in the range of *N* = 200–300, many of which are recruited from targeted schools (for an exception, see Simmonds, 2014). As a result, the current study sought to obtain a larger sample of educators (*N*~600) from a broad range of schools across the US through a citizen science website (TestMyBrain.org). This citizen science approach also allowed us to recruit a large sample of individuals from the general public (*N*~3,000). Our goal was to explore a variety of factors that might predict belief in myths, including demographics, educational attainment, and career and neuroscience-related exposure. We predicted that the most common neuromyths found in previous studies (i.e., right-brain/left-brain learners; learning styles) would also be prominent among US samples. We also predicted that the quality of an individual's neuroscience exposure would be related to belief in neuromyths, such that higher exposure to self-identified media might lead to more neuromyths, while higher exposure to academic neuroscience would lead to fewer neuromyths.

## Methods

### Participants

The neuromyths survey and associated demographic questionnaires were hosted on the website TestMyBrain.org from August 2014–April 2015. TestMyBrain.org is a citizen science website where members of the public can participate in research studies to contribute to science and learn more about themselves. In addition to these citizen scientists, we also explicitly advertised this study to individuals with educational or neuroscience backgrounds and encouraged them to visit TestMyBrain.org. Advertisements were distributed through professional and university listservs and social networks (i.e., Council for Exceptional Children, Spell-Talk, Society for Neuroscience DC chapter, American University (AU) School of Education, AU Behavior, Cognition, and Neuroscience program, AU College of Arts and Sciences Facebook page), as well as professional and personal contacts of the authors. In order to increase participation from educators, we used a snowball sampling technique in which each participant that completed the survey was asked to share the survey link with an educator that he/she knew through various social media (i.e., Twitter, Facebook, LinkedIn, email, etc.). This study was carried out in accordance with the recommendations of the Belmont Report as specified in US federal regulations (45 CFR 46). All participants gave written informed consent. The Institutional Review Board (IRB) at American University approved the research protocol for data collection and the IRB at the University of Denver determined that the research was exempt for de-identified data analysis.

The starting sample included surveys from 17,129 respondents worldwide. Only fully completed surveys were logged for further analysis. Participants were excluded if they reported experiencing technical problems with the survey (735 dropped), reported taking the survey more than once (e.g., based on a yes/no question at the end of the survey; 2,670 dropped), or reported cheating (e.g., looking up answers on the internet, discussing with an external person, 17 dropped).

Further exclusion criteria were age <18 years (2,121 dropped), missing data on gender or educational background (361 dropped), and non-US participant (according to IP address or self-endorsement; 6,899 dropped). Next, we plotted overall survey accuracy by time to complete the survey to filter individuals who might have rushed through the survey without reading the questions (22 dropped). On the demographics questionnaire, participants were asked if they were currently enrolled or completed middle school, high school, some college, college degree, or graduate degree. We excluded individuals with a middle school or high school degree from the analyses because of inadequate representation of these individuals in the sample (*N* = 424 general public, 3 educators, 0 high neuroscience exposure). Therefore, our analyses were limited to those with “some college” experience or beyond. These filtering and data cleaning steps brought our final sample size to *N* = 3,877 (*N* = 3,045 general public, 598 educators, 234 high neuroscience exposure).

One of the goals of this study was to compare the neuromyths performance of three groups: the general public, self-identified educators, and individuals with high neuroscience exposure. We defined “high neuroscience exposure” using a question on the demographics questionnaire which asked, “Have you ever taken a college/university course related to the brain or neuroscience?” Answer options for this item were “none,” “one,” “a few,” or “many.” Those who indicated “many” were categorized in the neuroscience group, unless they also reported being an educator, in which case they remained in the educator group (*N* = 53 educators also reported taking many neuroscience courses). We prioritized educator status over neuroscience exposure in this grouping in order to understand the full range of training backgrounds in the educator group. The “general public” group was composed of all individuals who completed the survey but did not self-identify as an educator or having high neuroscience exposure. We use the term “general public” to refer to the citizen scientists who participated, but we acknowledge the selection artifacts inherent to this group and address these limitations in the discussion section. Table 1 shows the demographic features of the three groups.

**Table 1:** Demographics.

	**General public (*N* = 3,045)**	**Educators (*N* = 598)**	**High neuroscience exposure (*N* = 234)**
Age	40.4 (16.9)^a^	44.9 (13.0)^b^	35.0 (13.3)^c^
Gender^*^	59% female	78% female	69% female
Education^*^			
Some college	37%	6%	12%
College	32%	15%	24%
Graduate	31%	79%	64%

abc *Values with different superscripts indicate significant differences, p < 0.001*.

* *chi-square indicates significant differences, p < 0.001*.

We examined differences among the three groups on basic demographic variables with one-way ANOVA and chi-square tests (Table 1). Age, gender, and highest level of education differed significantly between the groups and so were maintained as covariates in all subsequent analyses. Tables 2, 3 provide information about specializations in the educator and high neuroscience exposure groups.

**Table 2:** Specializations of educator subsample.

**Specialization**	***N***	**Percentage of educators^a^**
General education	185	31%
Early childhood (nursery, prek, kindergarten)	165	28%
Higher education (college/university)	161	27%
Special education	133	22%
Administrator (superintendent, dean, principal, director of program)	68	11%
Enrolled in teacher preparation program	33	6%
Gifted/talented	22	4%
Other (e.g., counselor, speech pathologist, tutor)	46	8%

a *A number of educators reported multiple specializations, so these groups are non-exclusive and do not sum to 100%*.

**Table 3:** Field of highest degree in individuals reporting high neuroscience exposure.

**Field**	**Percent**
Science	47%
Social Science	24%
Medicine	9%
Health and rehabilitation	6%
Education	3%
Humanities	3%
Nursing	2%
Engineering	2%
Law	1%
International	1%
Business	1%
Divinity	<1%
Arts	<1%
Public health	<1%

### Measures

#### Neuromyths survey

The web-based survey was adapted from Dekker et al. (2012) and consisted of 32 statements related to the brain and learning. In their survey, Dekker et al. (2012) designated 15 items as neuromyths and 17 items as general brain knowledge (e.g., “Learning occurs through changes to the connections between brain cells.”). We chose to investigate the factor structure of our adapted survey in order to determine which items would be categorized as “neuromyths” in our US-based sample.

Modifications from the survey by Dekker et al. (2012) were made to improve clarity for a US audience and to use language that is consistent with many widely held beliefs in the US. Substantive changes included revisions to the answer format from correct/incorrect to true/false. Additionally, neuromyth items that were worded to elicit a true response in Dekker et al. (2012) were reversed, such that a false response was now correct, except in one case to avoid a double-negative (#25, full survey is available in Table A1). Two questions were dropped and two questions were added in the current adaptation. A question about caffeine's effect on behavior was dropped following consultation with experts, who concluded that a simple true/false statement would not take into account the complexities of dosage. A question about fatty acid supplements (omega-3 and omega-6) affecting academic achievement was also dropped because of an emerging mixed literature on the effects of these supplements in ADHD (Johnson et al., 2009; Bloch and Qawasmi, 2011; Hawkey and Nigg, 2014). A question about the Mozart effect was added (#32) since this remains a prominent neuromyth in the US (Chabris, 1999; Pietschnig et al., 2010). A statement about dyslexia was added (#17) because of widespread misunderstanding about this neurodevelopmental disorder in the US (Moats, 2009). The appendix includes the modified questionnaire and intended answers. Items were presented in a different randomized order to each participant, and respondents were instructed to endorse each statement as either true or false. Only participants who answered all questions were included in analyses.

#### Demographics questionnaire

A demographics questionnaire was also included with questions about education background, career, neuroscience exposure, and science-related media exposure. Participants who reported having a career as an educator or within the field of education were directed to answer additional questions about their training, current employment, and specializations (i.e., special education, early education, higher education).

### Data cleaning and analysis

The data were analyzed using Stata/IC 14.0 for Windows. The statistical threshold was set to balance competing issues of the multiple comparison burden associated with 32 individual items on the survey and the expected correlation between the items. We opted to set the statistical threshold at *p* < 0.001 in order to account for the individual analyses of the 32 items on the neuromyth questionnaire (0.05/32 = 0.0015) and to adjust in the conservative direction for additional analyses of the composite neuromyth score.

An exploratory factor analysis (EFA) was conducted to examine the underlying relationships among the survey items. Because each survey item was dichotomous (correct or incorrect), a polychoric correlation matrix was used. Based on this EFA, a neuromyths factor score was constructed by summing the number of incorrect responses on the 7 neuromyth items that loaded on the first factor. For this factor, a high score indicated poor performance on neuromyths (i.e., more neuromyths endorsed).

Group comparisons between the general public, educators, and those with high neuroscience exposure were conducted for the neuromyths factor score and overall survey accuracy using one-way ANCOVAs, covarying for age, gender, and education (dummy coded with college as reference). Performance on individual neuromyth questions (true/false) was compared using logistic regressions covarying for age, gender, and education.

We were also interested in what factors predicted neuromyth performance in the full sample and in the subsample of educators. Ordinary least squares (OLS) and Poisson regressions were used to test the unique contribution of neuroscience exposure and exposure to science-related media, above and beyond the effects of age, gender, and education. In these regression analyses, categorical indicators (neuroscience exposure, career-related media, gender, and education level) were dummy-coded with reference categories indicated in **Tables 7, 8**.

We conducted Poisson regressions to analyze the variables predicting neuromyths because ordinary least squares regression (OLS) can give biased standard errors and significance tests for count data (Coxe et al., 2009). However, the coefficients of OLS can be more intuitively interpreted, so we also present the results from OLS multiple regressions, though we highlight the potential bias in the statistical significance of these results. Poisson regressions were appropriate because the neuromyths score was a count of incorrect items ranging from 0 to 7 that most closely resembled a Poisson distribution. Count variables are most appropriately analyzed using Poisson regression when the mean of the count is <10 (Coxe et al., 2009). In this study, the mean number of neuromyths endorsed was *M* = 4.53 (*SD* = 1.74). The Poisson distribution provided a good fit for the data [Pearson goodness of fit $X_{\left(3865\right)}^{2}$ = 2361.94, *p* = 0.99] and we did not find significant evidence of over-dispersion of the distribution (Likelihood ratio test of alpha, *p* = 0.99) indicating that the standard Poisson model was appropriate for this analysis.

Lastly, within the educator subsample, we conducted three additional regression analyses to examine the impact of specific specializations on neuromyth endorsement. We examined special educators and early educators as distinct specializations because we hypothesized that both groups might be exposed to more neuromyths: special educators because of their work with children with developmental disorders and early educators because of the prominence of birth-three myths about critical periods. We also examined those teaching in higher education as a distinct group with the hypothesis that these individuals might endorse fewer neuromyths. We made this prediction based on the fact that those in higher education would have easier access to evidence-based pedagogical resources (i.e., peer-reviewed journals, membership in national societies) and perhaps more interactions with neuroscience/psychology colleagues because of their college/university setting.

## Results

### Psychometrics

An exploratory factor analysis based on the polychoric correlation table, which was appropriate for our categorical (True/False) data, revealed three factors with eigenvalues >1. We rotated the factors using varimax rotation with Kaiser normalization (Kaiser, 1958). An orthogonal rotation, as opposed to an oblique rotation, was chosen because the three factors showed low correlations with each other (*r* = 0.04–0.21). Factor 1 had high factor loadings (>0.35) for seven “classic” neuromyths (#6, 8, 14, 17, 22, 26, and 32) (KR-20 = 0.63) related to learning styles, dyslexia, the Mozart effect, the impact of sugar on attention, the role of the right and left hemispheres in learning, and using 10% of the brain (Table 4). Items 24 and 29 (i.e., relationships between motor coordination and integrating right/left hemispheres and literacy) also loaded on factor 1, but we observed that those who reported having high neuroscience exposure also endorsed these items at very high rates, which led us to question whether the wording of these items led to confusion (i.e., perhaps respondents were thinking of the beneficial effects of exercise on cognition, generally (for a review see Hillman et al., 2008). We noted that those who reported having high neuroscience exposure performed at least 15% points better than the general public on all other items loading on factor 1, except for items 24 and 29. As a result, we dropped both items from the neuromyths factor and present the data for these items separately in **Table 6**. A neuromyths factor score was constructed by summing the number of incorrect items on the seven “neuromyths” items, such that higher scores on the neuromyths factor reflect a larger number of incorrect responses (i.e., endorsing more neuromyths). Further analyses used this neuromyths factor score to examine the prevalence and predictors of these “classic” neuromyths.

**Table 4:** Results from exploratory factor analysis.

**Short item description**	**Factor 1 “Classic” Neuromyths**	**Factor 2**	**Factor 3**	**Uniqueness**
14. Learning styles	0.84			0.27
26. Learning styles	0.76			0.36
8. Right-brain/left-brain learners	0.65			0.50
29. Motor coordination	0.55^*^			0.61
6. Use 10% of the brain	0.48			0.64
32. Classical music	0.48			0.71
17. Dyslexia	0.47			0.76
24. Motor coordination	0.41^*^			0.75
22. Sugar and attention	0.38			0.77
31. Brain shuts down during sleep		0.71		0.41
19. Mental capacity is genetic		0.55		0.70
1. Use our brains 24 h a day		0.53		0.71
28. New connections in brain/old age		0.48		0.71
13. Learning = addition of new cells		0.47		0.74
2. Native language before 2nd language		0.39		0.79
27. Education can't help LDs		0.39		0.83
5. Compensatory brain functions		0.34	0.37	0.72
10. Brain is developed by puberty		0.33		0.80
7. R/L hemispheres work together		0.31		0.79
25. Rehearsal of mental processes			0.48	0.70
15. Learning = new cell connections			0.45	0.78
20. Exercise and mental function			0.40	0.81
21. Enriched environment by age 3			−0.37	0.85
4. Brains shrink without water			−0.35	0.81
23. Adolescent circadian rhythms			0.35	0.86
30. Specific periods of learning			0.35	0.87
16. Breakfast and achievement			0.34	0.84
12. Information stored in cells				0.94
18. Birth and death of brain cells				0.94
3. Boys have bigger brains than girls				0.93
9. Brain development and gender				0.91
11. Critical periods of learning				0.89
Eigenvalue	4.34	2.47	1.48	
Percent of variance	33%	26%	20%	

*Factor loadings from rotated factor solution using Varimax rotation with Kaiser normalization (loadings < 0.3 are not listed for easier visualization)*.

* *Items dropped from factor 1 because individuals with high neuroscience exposure endorsed these items at very high rates*.

The second and third factors were less interpretable and theoretically coherent. Internal consistency for the items loading >0.35 on the two factors was quite low (KR-20 = 0.27–0.35). As a result, these factors were not retained for further analysis, but instead data for individual survey items are reported in **Table 6**.

### Group comparisons for neuromyths

Results from logistic regressions and ANCOVA analyses examining differences between groups on individual items and overall performance are presented in Tables 5, 6. Individuals in the general public endorsed significantly more neuromyths compared to educators and individuals with high neuroscience exposure. In turn, educators endorsed significantly more neuromyths than individuals with high neuroscience exposure (general public *M* = 68%, educators *M* = 56%, high neuroscience exposure *M* = 46%). A similar trend is present for the majority of the individual items that compose the neuromyths factor such that the general public endorsed the myths at the highest rate, followed by educators, followed by individuals with high neuroscience exposure who endorsed the myths at the lowest rate (Table 5).

**Table 5:** Neuromyth endorsement by group.

**Neuromyth factor 1 items (ranked by % incorrect)**	**Correct answer**	**General public (*N* = 3,045) (%)**	**Educator (*N* = 598) (%)**	**High neuroscience exposure (*N* = 234) (%)**
14. Individuals learn better when they receive information in their preferred learning style	FALSE	93^a^	76^b^	78^b^
26. Children have learning styles that are dominated by particular senses	FALSE	88^a^	71^b^	68^b^
17. A common sign of dyslexia is seeing letters backwards	FALSE	76^a^	59^b^	50^b^
32. Listening to classical music increases children's reasoning ability	FALSE	59^a^	55^ab^	43^b^
22. Children are less attentive after consuming sugary drinks and/or snacks	FALSE	59^a^	50^ab^	39^b^
8. Some of us are “left-brained” and some are “right-brained,” and this helps explain differences in learning	FALSE	64^a^	49^b^	32^b^
6. We only use 10% of our brain	FALSE	36^a^	33^a^	14^b^
Average percentage incorrect on neuromyths factor		68^a^	56^b^	46^c^

abc *Values with different superscripts are significantly different, p < 0.001 after covarying for age, gender, and education*.

**Table 6:** Accuracy on remaining survey items^*^.

**Remaining survey items (ranked by % incorrect)**	**Correct answer**	**General public (*N* = 3,045) (%)**	**Educator (*N* = 598) (%)**	**High neuroscience exposure (*N* = 234) (%)**
**ITEMS RELATED TO NEUROSCIENCE**				
3. Boys have bigger brains than girls, on average	TRUE	68^a^	69^a^	51^b^
4. Without sufficient water, students' brains shrink	FALSE	31^a^	35^a^	24^a^
5. When a brain region is damaged, other parts can take up its function	TRUE	26^a^	22^a^	12^b^
18. Normal brain development involves the birth and death of brain cells	TRUE	25^a^	21^ab^	13^b^
9. The brains of boys and girls develop at different rates	TRUE	21^a^	19^a^	29^a^
7. The left and right hemispheres work together	TRUE	21^a^	14^a^	8^a^
12. Information is stored in networks of cells distributed throughout the brain	TRUE	13^a^	13^a^	2^b^
25. Extended rehearsal of mental processes can change the structure of function of some parts of the brain	TRUE	10^a^	6^a^	5^a^
10. Brain development has finished by puberty	FALSE	8^a^	5^a^	3^a^
28. New connections in brain can occur in old age	TRUE	9^a^	6^a^	3^a^
1. We use our brains 24 h a day	TRUE	6^a^	4^a^	4^a^
31. When we sleep, the brain shuts down	FALSE	1^a^	1^a^	1^a^
**ITEMS RELATED TO LEARNING/EDUCATION**				
21. Children must be exposed to an enriched learning environment by age 3, or else learning capacities will be lost	FALSE	35^a^	39^a^	38^a^
2. It is best for children to learn their native language before learning a second language	FALSE	27^a^	18^a^	12^a^
11. There are specific periods in childhood after which certain things cannot be learned	FALSE	18*^a^*	19^a^	25^a^
23. Circadian rhythms shift during adolescence causing students to be tired during the first lessons at school	TRUE	29^a^	17^a^	21^a^
13. Learning is due to the addition of new cells to the brain	FALSE	17^a^	14^a^	10^a^
16. Academic achievement can be negatively impacted by skipping breakfast	TRUE	17^a^	13^a^	18^a^
15. Learning occurs through changes to the connections between brain cells	TRUE	14^a^	10^ab^	3^b^
27. Learning problems associated with developmental differences can't be improved by education	FALSE	12^a^	8^a^	9^a^
20. Vigorous exercise can improve mental function	TRUE	8^a^	7^a^	7^a^
19. Mental capacity is genetic and cannot be changed by environment or experience	FALSE	4^a^	3^a^	1^a^
30. There are specific periods in childhood when it's easier to learn certain things	TRUE	4^a^	4^a^	2^a^
**ITEMS RELATED TO MOTOR COORDINATION**				
29. Short bouts of motor coordination exercises can improve the integration of right and left hemispheres	FALSE	90^a^	89^ab^	81^b^
24. Exercises that rehearse coordination of motor-perception skills can improve literacy skills	FALSE	79^a^	80^a^	72^a^
Average percentage incorrect		24^a^	21^b^	18^c^

* *Shaded items were designated as neuromyths by Dekker et al. (2012), but did not load with the items on Factor 1*.

abc *Values with different superscripts are significantly different, p < 0.001 after covarying for age, gender, and education*.

The most commonly endorsed neuromyths across groups were related to learning styles and dyslexia. The most commonly endorsed neuromyths item was “individuals learn better when they receive information in their preferred learning style (e.g., auditory, visual, kinesthetic)” (general public *M* = 93%, educators *M* = 76%, high neuroscience exposure *M* = 78%). The second most commonly endorsed neuromyth was also related to learning styles “children have learning styles that are dominated by particular senses (i.e., seeing, hearing, touch)” (general public *M* = 88%, educators *M* = 71%, high neuroscience exposure *M* = 68%). Another common misconception was related to dyslexia: “a common sign of dyslexia is seeing letters backwards” (general public *M* = 76%, educators *M* = 59%, high neuroscience exposure *M* = 50%). It was interesting to note the very high rates of endorsement of these neuromyths even amongst individuals with high neuroscience exposure, though we note that these items are more closely related to the learning and special education fields than to neuroscience.

### Group comparisons for remaining survey items

The remaining survey items are grouped by topic areas (i.e., learning/education, brain/neuroscience, motor coordination/exercise) in Table 6. These survey items generally followed the same trend that was evident for the neuromyths, such that the general public performed least accurately and those with high neuroscience exposure performed most accurately, with educators falling in the middle (Table 6). Of these survey items which mostly tested factual brain knowledge, it is worth noting that 10% or fewer of those with high neuroscience exposure answered these items incorrectly, consistent with the alignment of their coursework with these factual neuroscience questions. This finding stands in contrast to the performance of the high neuroscience exposure group on the “classic” neuromyth items, where one-third to three-quarters of these individuals answered incorrectly depending on the item (with the exception of the 10% of the brain item, #6).

Two items (#24 and 29) involving motor coordination and exercise were the most frequently incorrect across groups. These items were endorsed at such high rates by the high neuroscience exposure group that we questioned whether the items were persistent neuromyths or whether the item wording was misunderstood by participants. Because of this ambiguity, we decided not to include these items on the neuromyths factor. After these two items, the most frequently incorrect item across groups was “Boys have bigger brains than girls, on average” (#3).

### Predictors of neuromyths

Results from both OLS multiple regression and Poisson regression are presented for the full sample and for the subsample of educators in Tables 7, 8. In both cases, the sum of incorrect items on the neuromyths factor was the outcome variable. Predictors included neuroscience exposure and science career-related media exposure, with age, gender, and education level as covariates.

**Table 7:** Regression results predicting neuromyths (i.e., % of items incorrect from neuromyths factor) in the full sample (*N* = 3,877).

	**Multiple regression**			**Poisson regression**	
	**B (SE)**	Beta^*^	***p*-value**	**B (SE)**	***p*-value**
Age	0.013 (0.002)	0.128	<0.001	0.003 (0.0005)	<0.001
Gender^a^	−0.300 (0.055)	−0.084	<0.001	−0.066 (0.016)	<0.001
Some college^b^	0.124 (0.068)	0.033	0.068	0.025 (0.019)	0.200
Graduate school	−0.615 (0.067)	−0.174	<0.001	−0.137 (0.020)	<0.001
1 neuroscience course^c^	−0.182 (0.071)	−0.041	0.010	−0.038 (0.021)	0.064
A few neuroscience courses	−0.260 (0.073)	−0.057	<0.001	−0.056 (0.021)	0.009
Many neuroscience courses	−1.022 (0.108)	−0.154	<0.001	−0.278 (0.037)	<0.001
Career websites^d^	−0.154 (0.061)	−0.043	0.011	−0.034 (0.018)	0.058
Career magazines	−0.042 (0.070)	−0.010	0.553	−0.010 (0.021)	0.642
Popular science magazines	−0.228 (0.060)	−0.059	<0.001	−0.051 (0.018)	0.004
Peer-reviewed science journals	−0.594 (0.073)	−0.136	<0.001	−0.148 (0.023)	<0.001
Adjusted *R*^2^		0.141			
*p*-value		<0.001			

* *Beta refers to the standardized beta estimate*.

a *Reference = female*.

b *Reference = college degree*.

c *Reference = no neuroscience courses*.

d *Reference = no career-related media exposure*.

**Table 8:** Regression results predicting neuromyths (i.e., % of items incorrect from neuromyths factor) in the educator sample (*N* = 598).

	**Multiple regression**			**Poisson regression**	
	**B (SE)**	Beta^*^	***p*-value**	**B (SE)**	***p*-value**
Age	0.014 (0.006)	0.093	0.022	0.004 (0.002)	0.036
Gender^a^	−0.114 (0.184)	−0.024	0.536	−0.030 (0.052)	0.556
Some college^b^	0.375 (0.369)	0.045	0.310	0.082 (0.092)	0.372
Graduate school	−0.612 (0.221)	−0.127	0.006	−0.140 (0.057)	0.015
1 neuroscience course^c^	−0.465 (0.195)	−0.099	0.018	−0.113 (0.054)	0.035
A few neuroscience courses	−0.706 (0.193)	−0.153	<0.001	−0.181 (0.055)	0.001
Many neuroscience courses	−1.300 (0.281)	−0.187	<0.001	−0.382 (0.090)	<0.001
Career websites^d^	−0.513 (0.164)	−0.130	0.002	−0.130 (0.046)	0.004
Career magazines	−0.025 (0.169)	−0.006	0.880	−0.004 (0.048)	0.930
Popular science magazines	−0.078 (0.175)	−0.017	0.657	−0.020 (0.049)	0.688
Peer-reviewed science journals	−0.723 (0.171)	−0.175	<0.001	−0.200 (0.050)	<0.001
Adjusted *R*^2^		0.148			
*p*-value		<0.001			

* *Beta refers to the standardized beta estimate*.

a *Reference = female*.

b *Reference = college degree*.

c *Reference = no neuroscience courses*.

d *Reference = no career-related media exposure*.

The results of the Poisson and OLS regressions were largely consistent. In cases of divergence, we deferred to the Poisson results which are most appropriate for the data distribution. For the full sample, demographic predictors of better performance on the neuromyths survey (*p* < 0.001) were age (being younger) and gender (being female). Education level also predicted neuromyth accuracy, such that those with a graduate degree performed better than those who finished college (Table 7). There was no significant difference between those who completed some college vs. those who obtained their college degree. Thus, only graduate education seemed to reduce the rate of neuromyth endorsement. Exposure to college-level neuroscience coursework also predicted neuromyths, such that those who reported completing many neuroscience courses performed better than those with no neuroscience courses. Individuals with 1 or a few neuroscience courses did not differ significantly from those with none, though there were nonsignificant trends (*p* = 0.064, *p* = 0.009, respectively). Exposure to science and career-related information also predicted neuromyths; specifically, those who reported reading peer-reviewed scientific journals performed better on neuromyths items (i.e., endorsed fewer myths) than those who did not engage with any career-related media. There were nonsignificant trends for those who read popular science magazines or visited career websites to perform better on neuromyths (*p* = 0.004; *p* = 0.058, respectively). Overall, the strongest predictors of lower rates of neuromyth endorsement in the full sample (determined by comparing standardized betas) were having a graduate degree, completing many neuroscience courses, and reading peer-reviewed journals. Although these were the strongest predictors, the effect sizes were modest. For instance, for the strongest predictor (graduate education) (partial η^2^ = 0.021), those with a graduate degree vs. a college degree endorsed less than 1 fewer item (out of 7) on the survey.

In the subsample of educators, significant predictors (*p* < 0.001) of neuromyths were similar to the full sample. Better performance was predicted by taking many neuroscience course and reading peer-reviewed scientific journals. Although these were the strongest predictors, the effect sizes were modest. For instance, for the strongest predictor (neuroscience courses) (partial η^2^ = 0.035), those who reported taking many neuroscience courses endorsed 1.3 items fewer on the survey (out of 7 total items) compared to those who had not taken any neuroscience courses.

In the educator subsample, we examined the impact of three specializations, special education, early education, and higher education. Each regression mirrored those in Tables 7, 8 (predictors for age, gender, education, neuroscience exposure, and science career-related media) except an additional predictor for specialization was added. In all three cases, the specialization was not associated with a significant difference in neuromyth performance in comparison to other educators without this specialization: special education (standardized β = −0.03, *p* = 0.45), early education (standardized β = −0.038, *p* = 0.33), and higher education (standardized β = −0.092, *p* = 0.06). The latter analysis for higher education was conducted only in those with graduate degrees (*N* = 471) because only a handful (*n* = 9) of individuals reported that they were teaching college but did not have a graduate degree. Although our results for higher education may suggest a trend for those in higher education to endorse fewer neuromyths, we note that this predictor does not meet our alpha threshold (*p* < 0.001) and its effect size is more modest than age, graduate school, and exposure to neuroscience and peer-reviewed science. Taken together, there was little support for educator specializations having a strong effect on neuromyth performance.

## Discussion

Neuromyths are frequently mentioned as an unfortunate consequence of cross-disciplinary educational neuroscience efforts, but there is relatively little empirical data on the pervasiveness of neuromyths, particularly in large samples from the US. Existing empirical data on neuromyths also exclusively focuses on educators, so an important question is whether educators' beliefs are consistent with the general public. One assumes that training in education and in neuroscience would dispel neuromyths, but it is unclear whether and to what extent this is the case. The goal of the current study was to establish an empirical baseline for neuromyth beliefs across three broad groups: the general public, educators, and individuals with high neuroscience exposure. Our results show that both educators and individuals with high neuroscience exposure perform significantly better than the general public on neuromyths, and individuals with neuroscience exposure further exceed the performance of educators. Thus, we find that training in both education and neuroscience (as measured by self-report of taking many neuroscience courses) is associated with a reduction in belief in neuromyths. Notably, however, both educators and individuals with high neuroscience exposure continue to endorse about half or more of the “classic” neuromyths, despite their training.

From the individual differences analyses, we found that the strongest predictors of neuromyths for the full sample were having a graduate degree and completing many neuroscience courses. For the subsample of educators, the strongest predictors were completing many neuroscience courses and reading peer-reviewed scientific journals. These findings suggest that higher levels of education and increased exposure to rigorous science either through coursework or through scientific journals are associated with the ability to identify and reject these misconceptions. As we hypothesized, the quality of the media exposure matters: peer-reviewed scientific journals showed the strongest association to neuromyths accuracy compared to other science-related media sources (i.e., websites, magazines). We discuss the implications of these results further below with an emphasis on implications for professional development and targeted educational programs addressing neuromyths.

### Clustering of neuromyths

Our analyses began with a psychometric investigation of our modified version of the widely-used neuromyths survey developed by Dekker et al. (2012). Results from an exploratory factor analysis revealed one factor consisting of seven core neuromyths (i.e., items about learning styles, dyslexia, the Mozart effect, the impact of sugar on attention, the role of the right and left hemispheres in learning, and using 10% of the brain). It was somewhat surprising to us that these “classic” neuromyths items clustered together because it seemed equally plausible that belief in one neuromyth would be independent from the other neuromyths. Nevertheless, the relationships among these neuromyths indicate that curriculum development should address several misunderstandings simultaneously because individuals who believe one neuromyth are likely to believe others as well. It is unclear why this might be the case, although one speculation is that a few misunderstandings about the complexity of learning and the brain will make one susceptible to a myriad of neuromyths. Alternatively, it is possible that these neuromyths are taught explicitly and simultaneously in some professional contexts. Regardless of the source, our data pointing to the clustering of neuromyths suggests that curricula must address multiple misunderstandings simultaneously, perhaps pointing out the connections between myths, in order to effectively address their persistence among educators.

One characteristic that seems to unite the 7 “classic” neuromyths that clustered together is an underestimation of the complexity of human behavior, especially cognitive skills like learning, memory, reasoning, and attention. Rather than highlighting these complexities, each neuromyth seems to originate from a tendency to rely on a single explanatory factor, such as *the* single teaching approach that will be effective for all children (learning styles) or *the* single sign of dyslexia (reversing letters), or *the* single explanation for why a child is acting out (sugar). Such over-simplified explanations do not align with the scientific literature on learning and cognition. For example, in the last 20 years, the field of neuropsychology has experienced a paradigm shift marked by an evolution from single deficit to multiple deficit theories to reflect an improved understanding of complex cognitive and behavioral phenotypes (Bishop and Snowling, 2004; Sonuga-Barke, 2005; Pennington, 2006). Such a multifactorial framework might be helpful for developing a curriculum that could dispel neuromyths. Within this framework, lessons would explicitly identify the *many* factors that influence learning and cognition and demonstrate how a misunderstanding of the magnitude of this complexity can lead to each neuromyth.

A curriculum to effectively dispel neuromyths should also include a preventative focus. For instance, lessons might make individuals aware of the cognitive bias to judge arguments as more satisfying and logical when they include neuroscience, even if this neuroscience is unrelated to the argument (McCabe and Castel, 2008; Weisberg et al., 2008). It might be particularly useful to point out that individuals with less neuroscience experience are particularly susceptible to this error in logic (Weisberg et al., 2008). A greater awareness of this cognitive error might be an important first step toward preventing new neuromyths from proliferating.

### Group comparisons: educators, general public, high neuroscience exposure

Our findings suggest that while teachers are better able to identify neuromyths than the general public, they still endorse many of the same misconceptions at high rates. These results are quite consistent with a parallel study conducted by Dekker et al. (2012) in a sample obtained from the UK and the Netherlands. Their study also reported that specific neuromyths showed a strikingly high prevalence among educators. Three of the neuromyth items that factored together in our study were asked with identical wording by Dekker et al. and so can be directly compared. For these items, our sample showed modestly better performance (learning styles (#14): Dekker-UK: 93% incorrect, Dekker-NL: 96%, US: 76%; sugar and attention (#22): Dekker-UK: 57%, Dekker-NL: 55%, US: 50%; 10% of brain (#6): Dekker-UK: 48%, Dekker-NL: 46%, US: 33%). Although performance was slightly better in the US sample, one likely explanation for this finding stems from the differences in recruitment between the studies. Dekker et al. (2012) used a school-based recruitment strategy where they invited all teachers in a school to participate, whereas the current study used online recruitment. Even in the context of these sampling differences, however, what is most notable is the degree of consistency in the prevalence of these neuromyths.

As expected, the high neuroscience exposure group endorsed the lowest number of neuromyths compared to educators and the general public, which is consistent with their background; however, they still endorsed about half (46%) of the neuromyths, on average, which might still be perceived as high. This result is even more striking in the context of the self-reported disciplinary affiliation of these individuals. Eighty-eight percent of the high neuroscience exposure group reported obtaining their highest degree in science, social science, medicine, health and rehabilitation, or nursing (47% science, 24% social science, 9% medicine, 6% health-related field, and 2% nursing), all fields that we would expect to have a strong neuroscience curriculum. Although we cannot be sure of the level and content of the neuroscience courses that the participants reported, it is striking that the vast majority of these individuals obtained their highest degree (88% college or graduate degrees) in a science- or health-related field, and yet the endorsement of neuromyths remained quite high.

One possible explanation for this surprising finding is to consider that the most commonly endorsed items by individuals with high neuroscience exposure were more closely related to learning and education than the brain and its function. This suggests that individuals with a significant number of neuroscience classes are susceptible to these misunderstandings because of the different levels of analysis of neuroscience vs. education. For instance, students might learn the synaptic basis of learning in a neuroscience course, but not educational theories of learning and how learning styles as an educational concept has developed and proliferated (see Pashler et al., 2008 for a discussion). Similarly, neuroscience students might learn the brain correlates of a developmental disorder such as dyslexia without discussing the behavioral research dispelling the neuromyth that it is caused by letter reversals. This finding suggests that if educators were to take a class in neuroscience that did not specifically address neuromyths, it would be unlikely to help with dispelling the misconceptions that are most closely related to learning and education.

Among all three groups, the two most prevalent neuromyths were related to learning styles theory and dyslexia (reversing letters). Endorsement of these neuromyths ranged from 76 to 93% in the general public group, 59 to 76% in the educator group, and 50 to 78% in the high neuroscience exposure group. Notably, over half of the surveyed teachers endorsed these items, which have direct implications for educational practice.

### Learning styles

Regarding learning styles theory, a meta-analysis from Pashler et al. (2008) reviewed findings from rigorous research studies testing learning styles theories and concluded that there was an insufficient evidence base to support its application to educational contexts. For example, the VAK (Visual, Auditory, Kinesthetic) learning styles theory posits that each student has one favored modality of learning, and that teachers should identify students' preferred learning styles and create lesson plans aimed at these learning styles (i.e., if a student self-identifies as a visual learner, content should be presented visually; Lethaby and Harries, 2015). The premise that visual learners perform better when presented with visual information alone than when presented with auditory information and vice versa for auditory learners is known as the “meshing hypothesis” (Pashler et al., 2008). While it is clear that many individuals have preferences for different styles of learning, Pashler et al. (2008) argue that the lack of empirical evidence for assessing these preferences and using them to inform instruction in order to improve student outcomes raises concerns about the pervasiveness of the theory and its near universal impact on current classroom environments. Willingham et al. (2015) argue that limited time and resources in education should be aimed at developing and implementing evidence-based interventions and approaches, and that psychology and neuroscience researchers should communicate to educators that learning styles theory has not been empirically validated (Willingham et al., 2015).

Several authors have discussed the lack of evidence for the VAK learning styles theory (Pashler et al., 2008; Riener and Willingham, 2010; Willingham et al., 2015), but in classroom practice, teachers have adapted the theory to fit classroom needs. For example, teachers weave visual and auditory modalities into a single lesson rather than providing separate modality-specific lessons to different groups of children based on self-identified learning style preferences. Hence, an unintended and potentially positive outcome of the perpetuation of the learning styles neuromyth is that teachers present material to students in novel ways through multiple modalities, thereby providing opportunities for repetition which *is* associated with improved learning and memory in the cognitive (for a review see Wickelgren, 1981) and educational literatures (for reviews, see Leinhardt and Greeno, 1986; Rosenshine, 1995).

Thus, the integration of multiple modalities can be beneficial for learning and this practice is conflated with the learning styles neuromyth. In other words, this particular neuromyth presents a challenge to the education field because it seems to be supporting effective instructional practice, but for the wrong reasons. To dispel this particular myth might inadvertently discourage diversity in instructional approaches if it is not paired with explicit discussion of the distinctions between learning styles theory and multimodal instruction. This specific challenge reflects the broader need to convey nuances across disciplinary boundaries of education and neuroscience to best meet the instructional and learning needs of students and educators. We would generally advocate for better information to dispel neuromyths that could be distributed broadly; however, the learning styles neuromyth appears to be a special case that requires deeper engagement with the educational community. For example, coursework or professional development might be the most effective way to address questions and controversies about the learning styles neuromyth. This stands in contrast to other neuromyths, such as that we only use 10% of our brain, which might be more easily dispelled by a handout with a short justification.

### Dyslexia and letter reversals

In contrast to the learning styles neuromyth, which might have unintended positive consequences, the neuromyth that dyslexia is characterized by seeing letters backwards is potentially harmful for the early identification of children with dyslexia and interferes with a deeper understanding of why readers with dyslexia struggle. This idea about “backwards reading” originates from early visual theories of dyslexia (Orton, 1925). Such visual theories of dyslexia were rejected decades ago as it became clear that impairments in language abilities, primarily phonological awareness, formed the underpinnings of dyslexia (Shaywitz et al., 1999; Pennington and Lefly, 2001; Vellutino et al., 2004). Some children with dyslexia do make letter reversals, but typically-developing children make reversals as well, particularly during early literacy acquisition (Vellutino, 1979). Such reversals early in literacy acquisition (i.e., kindergarten) are not related to later reading ability (i.e., 2nd-3rd grade) (Treiman et al., 2014). For children with dyslexia who make persistent letter reversals beyond the normative age, these reversals can best be understood as a *consequence* of poor reading and its associated cognitive impairments, rather than a cause of the reading problems. One prominent theory regarding the mechanisms underlying letter reversals posits that the reversals are the results of phonological confusion, rather than visual confusion (Vellutino, 1979). This research clarifies that the core deficit in dyslexia is not visual, yet this myth is remarkably persistent among educators (Moats, 1994; Washburn et al., 2014). One harmful effect of this neuromyth is evident in anecdotal reports from child assessment clinics where parents and teachers have delayed a referral for dyslexia because, though the child is struggling with reading, he/she is not reversing letters. Misunderstanding of the causal factors in dyslexia also leads to the persistence of visual interventions for reading that do not have an evidence base, and which may delay access to more effective phonologically-based treatments (Pennington, 2008, 2011; Fletcher and Currie, 2011). Efforts to educate teachers, parents, and medical professionals about the true underlying causes of dyslexia continue through national professional associations (i.e., American Academy of Pediatrics, 2009, 2014 and non-profit foundations like the International Dyslexia Association (i.e., Fletcher and Currie, 2011; Pennington, 2011).

### Limitations and future directions

Although these results provide an important empirical baseline for neuromyth prevalence in a broad US sample including educators, individuals with high neuroscience exposure, and the general public, the findings should be interpreted in light of the study limitations. First, our online recruitment strategy requires consideration of the generalizability of the sample. In this case, we primarily relied on volunteers who visited the TestMyBrain.org testmybrain.org website, some of whom were directed there by our emails to professional organizations for educators and neuroscientists. Hence, the sample was over-selected for individuals who have advanced education and who are already interested in science. Due to a limited number of participants who reported an education level as less than “some college,” we restricted our analyses to only those with some college or more. Therefore, the term “general public” may be misleading as our sample had higher rates of education than a nationally representative sample. In our sample, 100% of individuals had “some college” or more, 69% had a college degree or more, and 31% had a graduate degree, whereas these corresponding rates in a nationally representative sample are 59% (some college or more), 33% (bachelor's degree or more), and 12% (graduate degree) (Ryan and Bauman, 2016). The same critique is relevant to the educator sample where the rate of graduate education in our sample was 79% which exceeds the national average of 43% (Goldring et al., 2013). Although this selection is a limitation of the study, the fact that graduate education is associated with fewer neuromyths suggests that our results reflect the most optimistic case for the prevalence of neuromyths. We expect that the rate of neuromyths endorsement would only increase if a more representative population was obtained.

Another study limitation is related to our high neuroscience exposure group. Because we doubted the validity of self-report about the quality, content, and level of neuroscience courses, we opted for a simplified question: “Have you ever taken a college/university course related to the brain or neuroscience?” This item leaves many open questions about the nature of the neuroscience coursework, its specific emphasis, and what year it was taken. These variables are undoubtedly important to consider as there is certainly wide variation in quality and content of neuroscience curricula across the country, but would be time-intensive to collect with high validity (i.e., transcripts). A more realistic approach to control for variations in coursework would be to design studies using students who are enrolled in the same university neuroscience course.

Our online survey is subject to legitimate questions regarding quality control. We employed a number of procedures throughout the data cleaning process to ensure that we captured only legitimate responses, including examining the data for participants who were experiencing technical difficulties, cheating, taking the survey too quickly, and taking the survey more than once. Germine and colleagues have replicated well-known cognitive and perceptual experiments using the website used in the current study (TestMyBrain.org) with similar data cleaning techniques (Germine et al., 2012; Hartshorne and Germine, 2015). Although there is legitimate concern about noise with such an online approach, it is generally offset by the increase in sample size that is possible with such methods (Germine et al., 2012; Hartshorne and Germine, 2015). In this study, we note that sample sizes were largest for the educator and general public samples, while the individuals with neuroscience exposure was a smaller subgroup. Future research could benefit from further understanding of different disciplinary emphases within neuroscience training.

An additional limitation is that we did not have access to student outcomes, so we were unable to analyze how neuromyths endorsement among educators might influence academic performance in their students. Such data is important to consider in the context of our findings, as it is the only way to empirically determine whether or not believing neuromyths “matters” in the context of student outcomes. No studies to date have examined how teachers' endorsement of neuromyths may influence their attitudes toward their students' growth and learning. However, findings from previous research suggest that teachers' attitudes about the potential of students with learning disabilities to benefit from instruction predicts teachers' approaches to classroom instruction as well as students' achievement (Hornstra et al., 2010). Given this precedent, it is certainly possible that belief in neuromyths could impact teaching and student outcomes.

Lastly, we employed a slightly modified version of the neuromyths survey used by Dekker et al. (2012) because it has been widely used in a number of studies. Nevertheless, we did receive feedback from some participants that the true/false forced choice response was too simplistic. For example, the question “*There are specific periods in childhood after which certain things can no longer be learned”* is false in most cases, but some well-informed participants correctly pointed to areas of sensation and lower-level perception where this statement is true (i.e., Kuhl, 2010). This example demonstrates the limitation of a true/false paradigm for capturing complex scientific findings. Another limitation of the true/false paradigm is that we cannot discern participants' certainty of their answers because we did not include an “I don't know” answer choice. Future research should include such a choice or ask participants to rate their confidence in their answers on a Likert scale. Such information would be helpful for designing training curricula because a different approach might be necessary for individuals who are very sure that there are “right-brained” and “left-brained” learners, for example, compared to those who are unsure about this particular neuromyth.

### Summary

Although training in education and neuroscience predicts better performance on neuromyths, such exposure does not eliminate the neuromyths entirely. Rather, some of the most common myths, such as those related to learning styles and dyslexia, remain remarkably prevalent (~50% endorsement or higher) regardless of exposure to education or neuroscience. This finding is concerning because of the time and resources that many school districts may allocate toward pedagogical techniques related to these neuromyths that have very little empirical support. The findings reported in the current study create an opportunity for cross-disciplinary collaboration among neuroscientists and educators in order to develop a brief, targeted, and robust training module to address these misconceptions.

## Author contributions

LM developed the study concept and collaborated with LG on design and implementation. LM adapted the surveys for the current project. LG developed the platform for launching the survey and for collecting the web-based data. JC, AA, and LG provided consultation to LM regarding survey design and analysis. KM and LM performed the data analyses and planned the manuscript. KM wrote the first draft of the manuscript. KM and LM collaboratively revised the manuscript. LG, JC, and AA provided critical feedback on the manuscript and approved the final version.

### Conflict of interest statement

The authors declare that the research was conducted in the absence of any commercial or financial relationships that could be construed as a potential conflict of interest.

## Appendix A

**Table A1:** Brain Survey.

**#**	**Item**	**Answer**
1	We use our brains 24 h a day	True.
2	It is best for children to learn their native language before a second language is learned	False.
3	Boys have bigger brains than girls, on average	True.
4	If students do not drink sufficient amounts of water, their brains shrink	False.
5	When a brain region is damaged, other parts of the brain can take up its function	True.
6	We only use 10% of our brain.	False.
7	The left and right hemispheres of the brain work together	True.
8	Some of us are “left-brained” and some are “right-brained” and this helps explains differences in how we learn	False.
9	The brains of boys and girls develop at different rates	True.
10	Brain development has finished by the time children reach puberty	False.
11	There are specific periods in childhood after which certain things can no longer be learned	False.
12	Information is stored in the brain in networks of cells distributed throughout the brain	True.
13	Learning is due to the addition of new cells to the brain	False.
14	Individuals learn better when they receive information in their preferred learning style (e.g., auditory, visual, kinesthetic)	False.
15	Learning occurs through changes to the connections between brain cells	True.
16	Academic achievement can be negatively impacted by skipping breakfast	True.
17	A common sign of dyslexia is seeing letters backwards	False.
18	Normal development of the human brain involves the birth and death of brain cells	True.
19	Mental capacity is genetic and cannot be changed by the environment or experience	False.
20	Vigorous exercise can improve mental function	True.
21	Children must be exposed to an enriched environment from birth to three years or they will lose learning capacities permanently	False.
22	Children are less attentive after consuming sugary drinks and/or snacks	False.
23	Circadian rhythms (“body-clock”) shift during adolescence causing students to be tired during the first lessons of the school day	True.
24	Exercises that rehearse coordination of motor-perception skills can improve literacy skills	False.
25	Extended rehearsal of some mental processes can change the structure and function of some parts of the brain	True.
26	Children have learning styles that are dominated by particular senses (i.e., seeing, hearing, touch)	False.
27	Learning problems associated with developmental differences in brain function cannot be improved by education	False.
28	Production of new connections in the brain can continue into old age	True.
29	Short bouts of motor coordination exercises can improve integration of left and right hemisphere brain function	False.
30	There are specific periods in childhood when it's easier to learn certain things	True.
31	When we sleep, the brain shuts down	False.
32	Listening to classical music increases children's reasoning ability	False.

Adapted from Dekker et al. (2012).
